# Cardiac arrest in a newborn: A case of pseudohypoaldosteronism

**DOI:** 10.1002/ccr3.8265

**Published:** 2024-02-09

**Authors:** Kate A. Tauber, Kimberly Ermacor, James Listman

**Affiliations:** ^1^ Albany Medical Center Albany New York USA

**Keywords:** hyperkalemia, hypernatremia, neonate, pseudohypoaldosteronism

## Abstract

Pseudohypoaldosteronism (PHA) is a rare disease that can cause life‐threatening hyperkalemia, which could lead to cardiac arrest and death if not recognized and treated quickly. We report a case of a neonate who was diagnosed with PHA type 1 and found to have a novel variant gene mutation on the NR3C2 gene. A 5‐day‐old newborn presented in cardiac arrest with severe hyperkalemia, hyponatremia, and metabolic acidosis. Hypothermia treatment was initiated due to suspected hypoxic‐ischemic encephalopathy as well as electrolyte management with IV fluids and bicarbonate for the metabolic acidosis. Clinical suspicion and subsequent diagnostic testing led to a diagnosis of the renal form of PHA type 1. Genetic testing revealed a novel mutation on the NR3C2 gene of unknown significance (c.2891_2893dup plle964dup). The baby was discharged home on supplemental sodium and high‐calorie formula for catch‐up growth. Outpatient follow‐up is ongoing, and the dose of sodium supplement was slowly decreased and discontinued at 2 years. There is evidence for developmental delays which is likely secondary to the cardiac arrest although the MRI during hospitalization was noted to be within normal limits. Having a high clinical suspicion for pseudohypoaldosteronism is paramount to initiating treatment and preventing potential cardiac arrest and death in these patients. Novel mutations such as this one need to be further explored to determine their significance with this disease.

## Graphical abstract

This graph depicts the plasma levels of sodium (Na), potassium (K), and Creatinine (Cr) during the hospitalization of this infant diagnosed with pseudohypoaldosteronism.

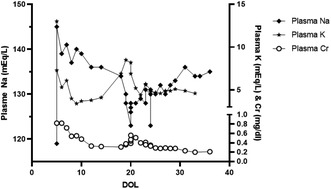

## 1 INTRODUCTION

Pseudohypoaldosteronism (PHA) is a rare syndrome caused by an apparent resistance to aldosterone. There are two different types of PHA, type 1 (PHA‐I) and type 2 (PHA‐II). PHA‐I can be inherited in an autosomal dominant (AD) or autosomal recessive (AR) manner. The AD form has been linked to mutations in the mineralocorticoid receptor and affects sodium transport in the principal cells of the kidney. The AR form is associated with mutations in the epithelial sodium channel (ENaC), which is expressed in multiple organs including the kidney, lung, colon, sweat glands, and salivary glands making this form more severe.^1^, ^2^ Therefore, the AD and AR forms are often referred to in the literature as the renal and systemic forms, respectively. The renal form often resolves within the first 2 years of life compared with the systemic form which is more severe and lifelong. PHA‐I typically presents in infancy with a clinical picture of salt wasting, failure to thrive, hyperkalemia, and acidosis, similar to symptoms found in infants with congenital adrenal hyperplasia.^3^ The volume depletion results from reduced sodium reabsorption by epithelial cells of the connecting segments and cortical collecting duct which, in turn, reduces the electrochemical gradient that normally favors the secretion of potassium and hydrogen ions. In addition to the renal dysfunction, the systemic form of PHA1 is also associated with respiratory infections and a positive sweat chloride test.^4^ The diffuse organ involvement makes the systemic form more lethal and newborns often present in cardiac arrest. PHA‐II has five different subtypes and is usually inherited in an AD manner, but there are some recessive cases in certain variants.^5^ This form of the disease results from an increase in the sodium‐chloride cotransporter in the distal convoluted tubule, which leads to volume expansion and hypertension. The volume expansion secondarily causes a hyporenin‐hypoaldosterone state similar to type 4 renal tubular acidosis. Thus, PHA‐II is not truly an aldosterone‐resistant state but biochemically appears that way.

Here, we report on a newborn who presented to an outside hospital emergency department in cardiac arrest with severe hyperkalemia. This patient was ultimately diagnosed with the renal form of PHA1 with a genetic mutation of uncertain significance on the gene associated with PHA1.

## 2 CASE PRESENTATION

A five‐day‐old female newborn, a product of an uncomplicated pregnancy and delivery, presented to an emergency department in cardiorespiratory arrest of unknown etiology. She was hypothermic and had poor respiratory effort requiring intubation. She had a profound mixed metabolic/respiratory acidosis with a pH < 6.70 and base deficit of −30 mmol/L. Multiple doses of epinephrine, atropine, sodium bicarbonate, and normal saline were given. Therapeutic hypothermia treatment was initiated on transport to our neonatal ICU due to suspected hypoxic‐ischemic encephalopathy.

Admission laboratories showed a sodium level of 131 mEq/L, a potassium level of >9 mEq/L, and a bicarbonate level of 10 mmol/L. These levels normalized after repeated normal saline, sodium bicarbonate, insulin, and calcium gluconate boluses. Other abnormal lab values included elevated AST and ALT levels (3087 and 1768 IU/L, respectively), elevated troponin of 0.7 ng/mL, and elevated lactate dehydrogenase (>10,000 hemolyzed, then 6917 IU/L). Her blood urea nitrogen and serum creatinine levels were mildly elevated at 37 and 1.0 mg/dL, respectively.

She also presented in disseminated intravascular coagulation and received multiple transfusions. By her second day after admission, her clotting studies normalized. An infectious workup including bacterial and viral blood and CSF cultures and a urine culture were all negative. She received antibiotics for a total of 10 days for presumed sepsis.

Therapeutic hypothermia treatment was initiated on transport and continued for 72 h per protocol. Her initial neurological examination revealed generalized hypotonia, mildly responsive pupils, and an absent gag, suck, root, and grasp reflex. A brain CT scan revealed signs of anoxic injury and brain edema. She was loaded with an anticonvulsant on her second day of admission due to concern for seizure activity. Maintenance dosing was discontinued after a continuous EEG showed no seizure activity. Her neurologic examination gradually improved in relation to her tone, alertness, and reflexes. An MRI on day of life 11 did not reveal any signs of hypoxic brain injury. She was extubated on day of life 12 and weaned off all respiratory support by day of life 17.

**FIGURE 1:**
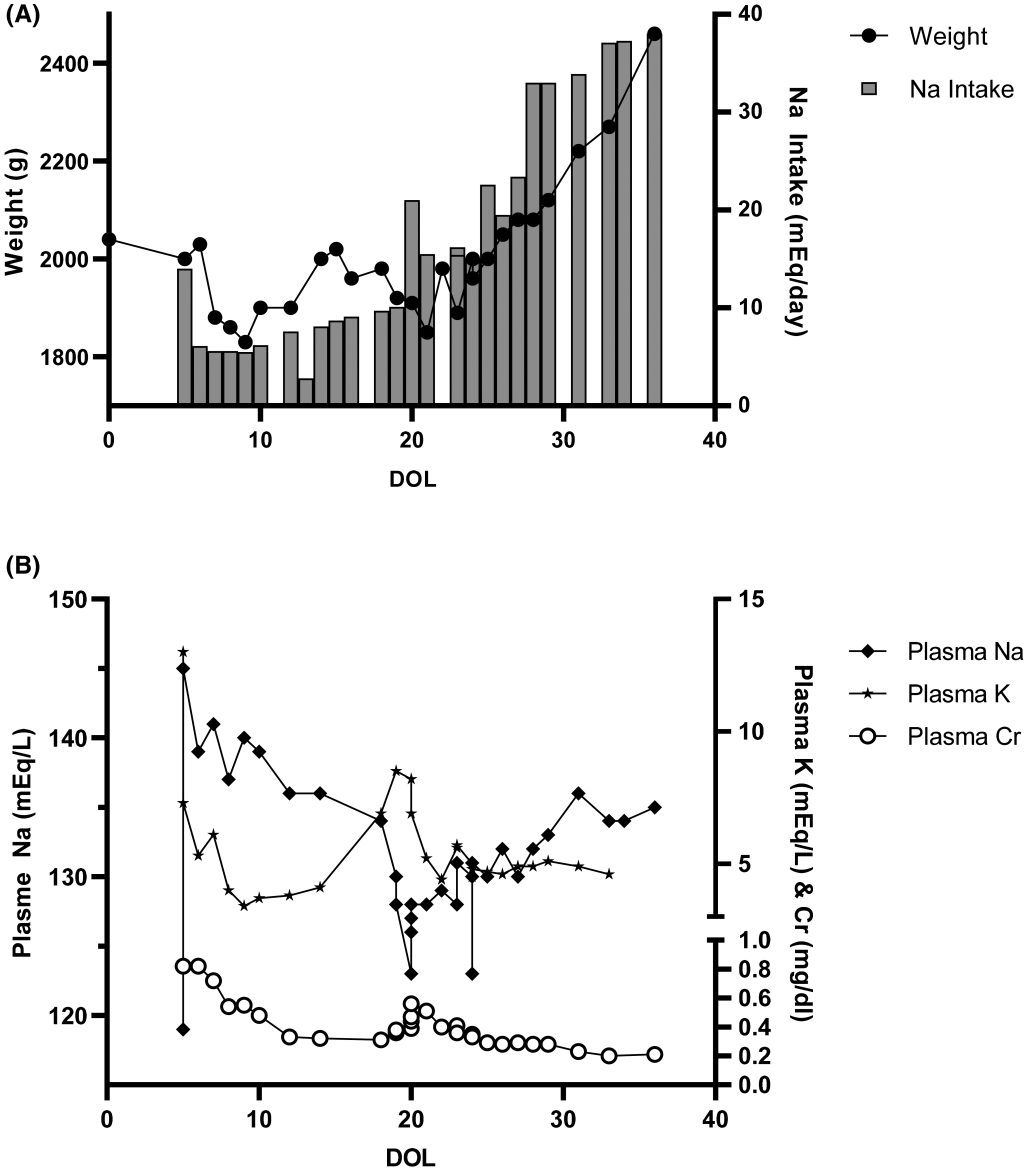
A. Graph of the weight and sodium intake over the course of the hospitalization. B. Graph depicting the plasma levels of sodium (Na), potassium (K), and creatinine (Cr) during the hospitalization.

Oral feeds were started and she was taken off all IV fluids by day of life 16. Repeated serum electrolytes off IV fluids revealed progressive development of hyponatremia (nadir of 123 mEq/L) and hyperkalemia (peak of 8.5 mEq/L) by day of life 20 (Figure 1). There was no evidence of metabolic acidosis. Urine studies revealed elevated urine sodium excretion (sodium 80 mEq/L, potassium 18 mEq/L, urine osmolality 279 mOs/kg with a serum osmolality 274 mOs/kg). Intravenous fluids with sodium chloride were restarted, and her formula was changed to one without added solute or added calories. At this point, a salt‐wasting condition associated with impaired potassium secretion was suspected. On day of life 19, a serum cortisol (22.4 ug/dL) was normal suggesting that she did not have congenital adrenal hyperplasia (CAH; confirmed by negative newborn screening). Despite this, a trial of fludrocortisone was given but without effect. Renin and aldosterone levels later returned elevated at 131 ng/mL/h and 2010 ng/dL, respectively, confirming a suspicion that she had pseudohypoaldosteronism. Sodium administration both in IV and oral forms was titrated upwards to ensure weight gain, normal GFR, and normalization of her plasma sodium and potassium over the following weeks. She was discharged to home on her 38th day of life on enteral sodium supplementation of 14 mEq/kg/day.

At 4 months of age, she underwent sweat chloride testing that was normal which ostensibly ruled out the systemic form of the disease. Genetic testing revealed a mutation of unknown significance in the NR3C2 gene (c2891‐2893dup (p. Ile964dup)). Over the next 2 years, her sodium supplements were gradually weaned off and her electrolytes have remained normal. At 4 years of age, she does not have any significant motor delays but does have moderate speech delays and some hearing impairment.

## 3 DISCUSSION

Common causes of hyperkalemia in an infant include acute hemolysis, rhabdomyolysis or redistribution of potassium out of muscle (usually associated with shock status), renal disorders, and hormonal disorders.^6^ Many of these may be suspected upon historical grounds and the initial interpretation that this patient's hyperkalemia was due to shock was premature. Oddly, the classic findings suggesting a problem within the renin aldosterone pathway took about 12 days after presentation to manifest itself and we can only speculate as to why this was the case. First, the baby received a large amount of saline in the first day during her resuscitation and the extracellular space was most certainly overfilled. Therefore, the rapid loss of weight due to diuresis following the first day of admission probably reduced the baby to near her dry weight, not below it. Furthermore, her glomerular filtration rate was still low for several days after presentation, which would be protective to her body's sodium content. Lastly, she was not receiving potassium until she started feeding several days later. Once she became hyponatremic and hyperkalemic between days of life eighteen and twenty, it became apparent that there was something underlying her electrolyte abnormalities. PHA and CAH can present in similar ways and were high on the list of potential diagnoses for this patient. However, CAH only rarely has been reported to cause cardiac arrest and we quickly ruled it out by lack of virilization, her normal cortisol level, and normal newborn screen. This made us suspicious for PHA which was ultimately verified once her high renin and aldosterone levels came back.

Besides the genetic forms of PHA mentioned above, it should be noted that there are secondary causes of PHA as well including various forms of congenital renal disease associated with obstruction or reflux, certain medications (like bactrim) used in the face of chronic kidney disease, and pyelonephritis, particularly in infants.^7^ Ironically, Type II Bartter syndrome, which is typically associated with hypokalemia, has been reported to cause transient hyperkalemia in the newborn period.^8^ Of all these causes, perhaps systemic PHA due to defects in the epithelial sodium channel is most prone to cause severe electrolyte abnormalities because the defect extends outside the kidneys to include the skin, respiratory tract, and colon where sodium losses can be exacerbated by insensible losses or GI losses and the colon's role in excretion of a small portion of the daily potassium load is impaired.^9^

Since 1958, when PHA was first described, there have been numerous case reports and novel mutations described.^10^ However, there have been no genotype/phenotype correlations between the mutations described and the clinical symptoms the patients exhibited.^11^, ^12^ The patient in this case was found to have a mutation of unknown significance in the NR3C2 gene (c2891‐2893dup (p. Ile964dup)). The NR3C2 gene codes for the mineralocorticoid receptor in the kidney and is responsible for the binding of aldosterone.^13^ Mutations in this region result in the inability of aldosterone to bind to its receptor leading to increased salt loss and potassium retention. Despite the mutation, this form is typically transient and treatment with high‐dose sodium supplements is only required for the first few years of life. The gradual resolution of symptoms over time is thought to be a result of the change in diet from low sodium breastmilk to higher sodium diet with solid foods as well as the maturation of sodium reabsorption function over time.^14^ We did not think it necessary to formally test sodium channel genes because her sweat chloride test functionally ruled out the systemic form of the disease and her relatively benign course after discharge did not fit the severe phenotype.

The mutation seen in this patient falls in the AF‐2 segment of the ligand binding domain (LBD), a highly conserved region of both glucocorticoid and mineralocorticoid receptors.^15^ Following ligand binding, the LBD undergoes rearrangement and exposes the AF‐2 region, which is instrumental for interaction with transcriptional coactivators. Experimental evidence shows that mutation of the adjacent residue at position 962 reduces the transcriptional activity of the gene due to reduced interaction with coactivator at the AF‐2 region.^16^ Therefore, we hypothesize that our patient's duplication of ILE at position 963 could affect the length of Helix 12 of the LBD and interferes with coactivator binding or disrupts the integrity of the LBD structure and is the cause of her phenotype.

In conclusion, PHA is a rare disorder. The clinical course depends on whether the mutation is in the mineralocorticoid receptor (renal form of PHA‐I) or in one of the subunits of the epithelial sodium channel (systemic form of PHA‐I). Both types present with severe and often life‐threatening hyperkalemia, hyponatremia, and metabolic acidosis. Different from CAH, they will have normal 17‐OHP levels and high plasma levels of aldosterone and renin. Clinical suspicion of PHA is paramount to begin aggressive treatment with IV hydration, sodium and bicarbonate supplementation, and correction of hyperkalemia. Daily enteral salt supplementation will be necessary but can be titrated down over the first 1–2 years of life in the renal form. The relationship between the various reported mutations and clinical outcomes is not yet known, but hopefully with advances that have been made in the field of genetic testing we may soon have an answer.

## AUTHOR CONTRIBUTIONS

**Kate A. Tauber:** Conceptualization; formal analysis; methodology; writing – original draft; writing – review and editing. **Kimberly Ermacor:** Data curation; writing – review and editing. **James Listman:** Conceptualization; formal analysis; methodology; writing – review and editing.

## CONFLICT OF INTEREST STATEMENT

The authors declare no conflicts of interest.

## Data Availability

The data that support the findings of this study are available on request from the corresponding author. The data are not publicly available due to privacy or ethical restrictions.
